# Morphofunctional Alterations in Zebrafish (*Danio rerio*) Gills after Exposure to Mercury Chloride

**DOI:** 10.3390/ijms18040824

**Published:** 2017-04-13

**Authors:** Rachele Macirella, Elvira Brunelli

**Affiliations:** Department of Biology, Ecology and Earth Science, University of Calabria, Via P. Bucci 4/B, 87036 Rende (Cosenza), Italy; rachele.macirella@unical.it

**Keywords:** inorganic mercury, gills, acute effects, histology, ultrastructure, confocal microscopy, RT-PCR, metallothionein, Na^+^/K^+^-ATPase

## Abstract

Mercury (Hg) is a global pollutant that may exert its toxic effects on living organisms and is found in both aquatic and terrestrial ecosystems in three chemical forms; elemental, organic, and inorganic. The inorganic form (iHg) tends to predominantly accumulate in aquatic environments. The gill apparatus is a very dynamic organ that plays a fundamental role in gas exchange, osmoregulation, acid-base regulation, detoxification, and excretion, and the gills are the primary route of waterborne iHg entrance in fish. In the present work we investigated the morphofunctional and ultrastructural effects in *Danio rerio* gills after 96 h exposure to two low HgCl_2_ concentrations (7.7 and 38.5 µg/L). Our results clearly demonstrated that a short-term exposure to low concentrations of mercury chloride resulted in gill morphology alterations and in the modifications of both Na^+/^K^+^-ATPase and metallothioneins (MTs) expression pattern. The main morphological effects recorded in this work were represented by hyperplasia and ectopia of chloride cells (CCs), lamellar fusion, increased mucous secretion, alteration of pavement cells (PVCs), detachment of the secondary epithelium, pillar cell degeneration, degeneration, and apoptosis. Trough immunohistochemistry and real-time PCR analysis also showed a dose-related modulation of Na^+^/K^+^-ATPase and MTs.

## 1. Introduction

Mercury (Hg) is a highly toxic heavy metal that has no known vital or beneficial function for living organisms [1]. Despite being a natural constituent of the earth’s crust, human activities, such as mining, precious metal extraction, industry, and agriculture have altered its geochemical cycles and environmental distribution. Mercury is widely distributed in both aquatic and terrestrial ecosystems in three chemical forms; elemental, organic, and inorganic. The inorganic form (such as mercury chloride) tends to predominantly accumulate in aquatic environments [2].

Whatever the source, natural or anthropogenic, mercury hazards are related to its high tendency for bioaccumulation and toxicity. The risk posed by this heavy metal for fish and wildlife has been underestimated from the beginning. Mercury drew international attention in the 1950s and 1960s, after realizing that the poisoning, and in some cases the death, of many people in Japan was due to the consumption of mercury-contaminated fish and seafood from Minamata Bay [3]. Later, the strong evidence of mercury impact on both the environment and human health has highlighted the global concern of mercury contamination, and in 2005 mercury was ranked third in the Comprehensive Environmental Response, Compensation, and Liability Act (CERCLA) Priority List of Hazardous Substances [2].

Fish are considered good bioindicators [4,5] due to their ability to very quickly absorb chemicals through their gills and their body surface or by direct ingestion, thus making them highly sensitive to environmental pollutants [6].

In particular, the gills provide the most extensive surface exposed to the aquatic medium and it is the first organ affected by toxic substances [7,8,9,10]. The gill apparatus is a very dynamic organ that plays a fundamental role in gas exchange, osmoregulation, acid-base regulation, detoxification, and excretion [11,12]. Moreover, the gills are the primary route of waterborne inorganic mercury (iHg) entrance in fish [13,14,15]. Recently, it has been demonstrated in *Diplodus sargus* that the accumulation level of iHg in gills is higher and faster than in other tissues, such as liver, brain, and blood, when the fish were exposed via water [16]. The iHg uptake involves several mechanisms that are both active and passive, affecting ion transport processes and others [15,17,18,19].

Although the inorganic mercury is the most common form of mercury in water and in spite of its toxicity [20,21], data on iHg are surprisingly scarce compared with methyl mercury (MeHg), and the iHg effects have been largely under-investigated.

In drawing the outline of sub-lethal effects induced by heavy metals, a wide range of biological responses can be used as biomarkers [22]. Histopathological studies are commonly used when analyzing the toxic effects of heavy metals and provide a powerful tool to quickly evaluate the health and fitness of individuals [23]. Besides histopathology, many kinds of enzymes are considered as sensitive biochemical markers for metal contamination.

In freshwater fish an acute exposure to sub-lethal concentrations of iHg can disrupt gill functions by inducing morphological alterations [24,25,26,27,28] and/or through the disruption of Na regulation (also as a consequence of the alteration of Na^+^/K^+^-ATPase activity) [15,17]. As suggested by Poopal and colleagues [29], since the modulation of Na^+^/K^+^-ATPase activity precedes the appearance of gross osmoregulatory dysfunction, this enzyme can represent an early warning of pollution [29,30,31]. These findings lead to the use of the Na^+^/K^+^-ATPase pump as a powerful biomarker of osmotic stress after exposure to iHg.

Metallothionein (MTs) are low molecular weight proteins with a strong affinity for several metals that are typically involved in the regulation of essential metals (such as copper/Cu and zinc/Zn) and in the cellular detoxification of non-essential metals such as Hg, cadmium (Cd), and silver (Ag) [32]. Furthermore, these proteins confer resistance to organisms that live in environments contaminated by heavy metals [33,34]. Due to these characteristics, the metallothionein (MTs) are typically used to evaluate the impact of heavy metals in aquatic organisms [32,35,36,37].

Zebrafish (*D. rerio)* has become a model organism widely used in eco-toxicological studies. The numerous advantages for the use of zebrafish are proven by the growing number of studies using this organism and comprise its small body size, easy husbandry, and early morphology [38]. Moreover, zebrafish is particularly important to evaluate toxic effects induced by metal contamination because of the high soft water tolerance of this species [39].

In view of this background, our research was designed to investigate the morpho-functional effects induced by iHg on the zebrafish gill apparatus, one of the key compartments of fish. The present study is part of a more comprehensive study aimed to document the effects of mercury chloride on zebrasfish. We assessed the morphological and ultrastructural changes of gills associated with an acute exposure to two sub-lethal concentrations of mercury chloride (HgCl_2_). Furthermore, we used a real-time PCR (RT-PCR) for the detection and quantification of Na^+^/K^+^-ATPase and metallothioneins genes, and a confocal laser scanning microscope for immunolocalization of these proteins under both basal and experimental conditions.

The effects induced by mercury on the gill apparatus have been reported in several species of fish, shellfish, and crustaceans [25,26,40,41], but no one has reported the effects induced by this heavy metal on *D. rerio* gills through a morpho-functional approach. Only one study conducted by Glynn and his colleagues [18] analyzed the accumulation of inorganic mercury and cadmium in zebrafish gills. To our knowledge this is the only report on the histological, ultrastructural, and functional effects induced by iHg on *D. rerio* gills.

## 2. Results

### 2.1. Structure and Ultrastructure

#### 2.1.1. Control

The morphology of the gills in *D. rerio* is similar to that of other freshwater Teleostei as reported in detail by Karlsson [42]; only a brief general description relevant to the present paper will be given. From a histological and ultrastructural point of view, the gill apparatus of *D. rerio* revealed no significant differences between males and females.

**Figure 1:**
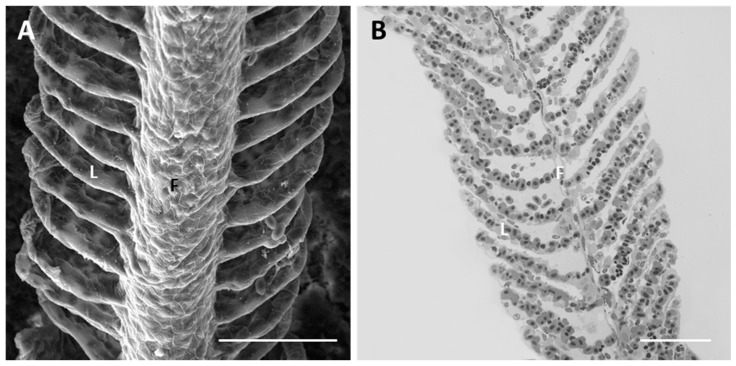
*D. rerio* gill apparatus in basal conditions: (**A**) SEM micrographs showing concentric microridges of pavement cells (PVCs). F = filament and L = lamellae. Bar 50 µm; (**B**) light micrographs in toluidine blue showing general morphological organization of branchial epithelium; F = filament and L = lamellae. Bar 50 µm.

Each gill is supported by four branchial arches which provide insertion to a double series of filaments (i.e., primary filaments or main filaments). From each filament departs a double series of secondary lamellae (i.e., secondary filaments) characterized by a large flattened surface (Figure 1A,B).

Under SEM, we can recognize the polygonal pavement cells (PVCs) that form the external epithelial layer in both the filament and lamellae. PVCs show well-outlined boundaries and are equipped with numerous concentric microridges at their apical surface (Figure 1A).

**Figure 2:**
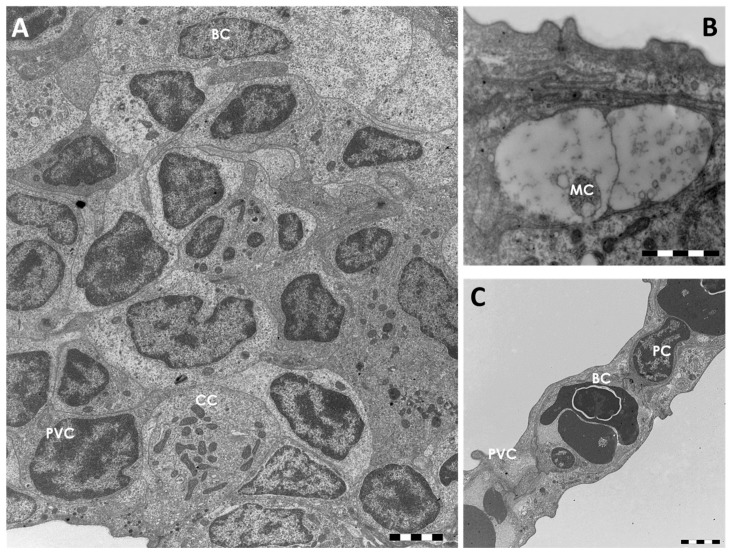
TEM micrographs of the gill apparatus in *D. rerio* under basal conditions: (**A**) cellular organization in the primary epithelium. CC = chloride cell, PVC = pavement cell; BC = basal cell; (**B**) high magnification of a mucous cell (MC); (**C**) ultrastructural organization of the secondary epithelium. PVC = pavement cell; BC = basal cell; PC = pillar cell. All bars 2 µm.

Histological observations allow us to identify, in the primary epithelium, four cell types; pavement cells (PVCs), mucous cells or goblet cells (MCs), chloride cells (CCs) (also called mitochondria-rich cells), and basal cells (Figure 2A). Undifferentiated basal cells originate at the innermost layer that makes contact with the underlying basal membrane (Figure 2A). Both CCs and MCs were scattered beneath the PVCs, in particular in the interlamellar region. Their apical surface is partially covered by adjacent PVCs and open outwards through small pores (Figure 2A,B).

The MCs are easily identifiable by their roundish shape and by the presence of numerous electron clear granules that filled the entire cytoplasm (Figure 2B). The CCs are large round or ovoid cells characterized by numerous mitochondria and by a complex tubulo-vesicular system in their cytoplasm (Figure 2A). The secondary epithelium is very simple and it is composed by an external layer of PVCs and an inner layer of basal cells (Figure 2C). The pillar cells that regulate capillary blood flow and endothelial cells are easily recognizable (Figure 2C).

#### 2.1.2. Exposed Fish

**Figure 3:**
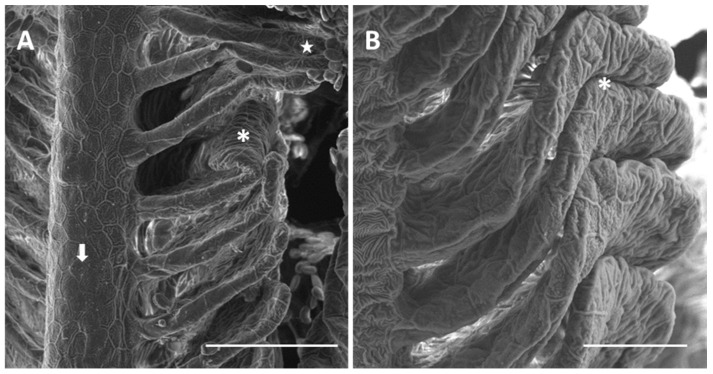
SEM micrographs of the primary and secondary epithelium in *D. rerio* after 96 h of exposure to 7.7 µg/L of HgCl_2_: (**A**) degeneration in PVCs microridges in the primary epithelium (arrow); folding in the distal portion of lamellae (asterisk) and lamellar fusion (star). Bar 50 µm; (**B**) higher magnification of lamellar fusion (asterisk). Bar 20 µm.

Exposure to 7.7 µg/L of mercury chloride (we will refer to 7.7 µg/L as the “low” concentration) induces severe morphological and ultrastructural changes in the gill apparatus of *D. rerio*. Under SEM we can note that in some areas of the primary epithelium, the typical microridge arrangement of the PVCs is missing (Figure 3A). The secondary lamellae appear folded and fused in the distal portion (Figure 3A,B), thus occluding the interlamellar space.

**Figure 4:**
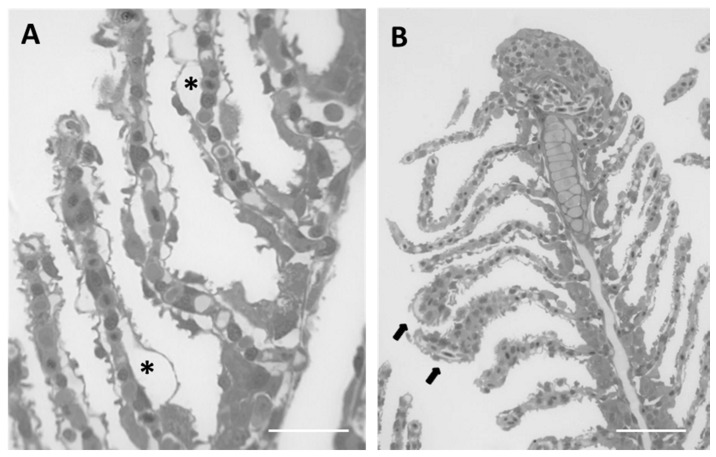
Sagittal section in toluidine blue of the gill apparatus in *D. rerio* after 96 h of exposure to 7.7 µg/L of HgCl_2_: (**A**) detachment of epithelium from connective tissue that create wide lacunae in gills lamellae (asterisk). Bar 20 µm; (**B**) hypertrophy in endothelial cells and blood congestion (black arrow). Bar 50 µm.

The main alterations in the gills can be detected in the secondary lamellae. Histological analysis reveals an extensive detachment of the epithelium from the connective tissue thus originating wide lacunae within lamellar tissues. The hypertrophy of epithelial cells is particularly evident in the distal portion of lamellae leading to folding and, in some cases, to lamellar fusion (Figure 4A,B). Hypertrophy of endothelial cells along with blood vessel congestion are also noticed (Figure 4B).

**Figure 5:**
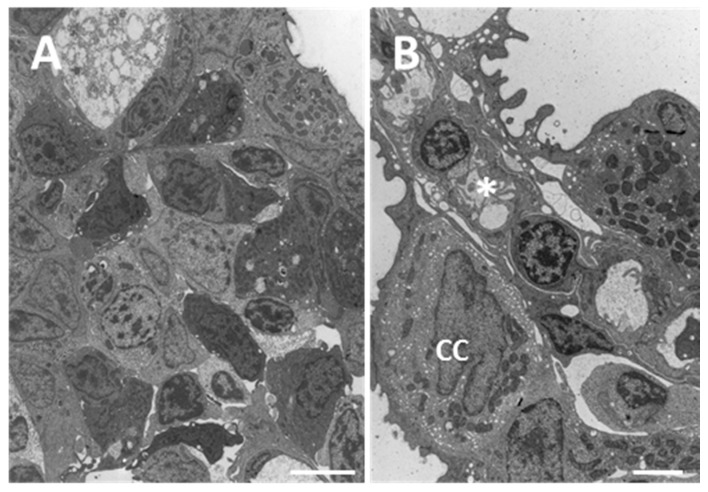

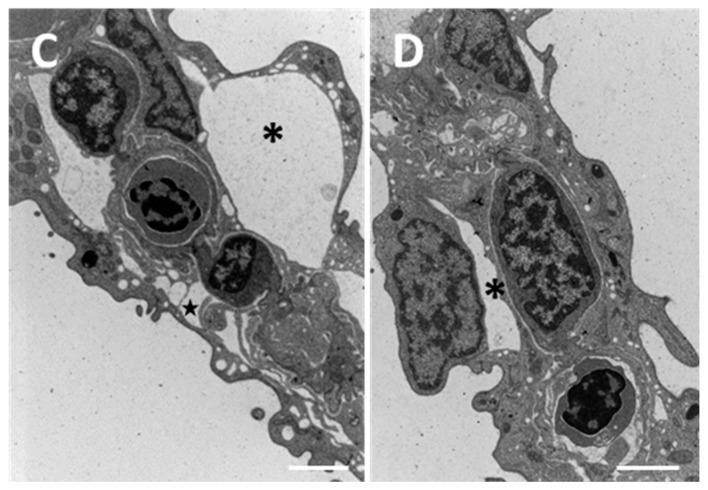
TEM micrographs of the gill apparatus in *D. rerio* after 96 h of exposure to 7.7 µg/L of HgCl_2_: (**A**) early degeneration in the inner layers of filaments; (**B**) detachment of epithelium (asterisk) and hypertrophy of chloride cell (CC); (**C**) high magnification of detachment in secondary epithelium (asterisk) and degeneration of PVCs (star); (**D**) loss of PVCs connections with the basal lamina (asterisk). All bars 2 µm.

Despite the well maintained gross morphology, ultrastructural observation of the main epithelium reveals the appearance of degenerative phenomena; apoptotic and necrotic cells as well as macrophage infiltration occur and in some cases completely degenerated cells could be seen (Figure 5A). In the interlamellar area, CCs appear hypertrophic and broaden out towards the secondary epithelium (Figure 5B). Moreover, in the secondary lamellae the complete detachment of the epithelium from the basal lamina is evident (Figure 5B–D) and the cytoplasm of PVCs appears highly vacuolated (Figure 5C,D).

**Figure 6:**
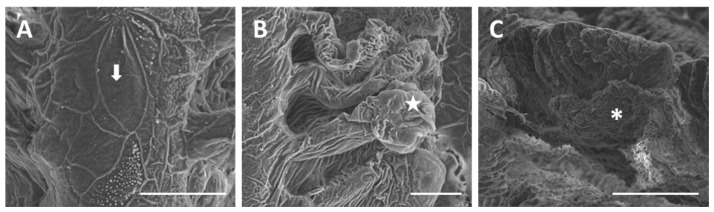
SEM micrographs of the gill apparatus in *D. rerio* after 96 h of exposure to 38.5 µg/L of HgCl_2_: (**A**) degeneration of microridges in primary epithelium (arrow). Bar 20 µm; (**B**) wrinkled surface of primary and secondary epithelium with swelling in the distal portion of lamellae (star). Bar 20 µm; (**C**) increase in mucous secretion (asterisk). Bar 100 µm.

Gill alterations in *D. rerio* are more conspicuous after 96 h of exposure to 38.5 µg/L of HgCl_2_ (we will refer to 38.5 µg/L as the “high” concentration). SEM observations on the main filament show that the microridges of PVCs completely lose their typical arrangement and, in some cases, disappear (Figure 6A). The epithelial surface, of both filaments and lamellae, appears wrinkled and almost all the secondary lamellae show an intense swelling in their distal portion (Figure 6B). At this concentration it is also relevant that the presence of a strong mucous blanket covers the entire gill apparatus (Figure 6C).

**Figure 7:**
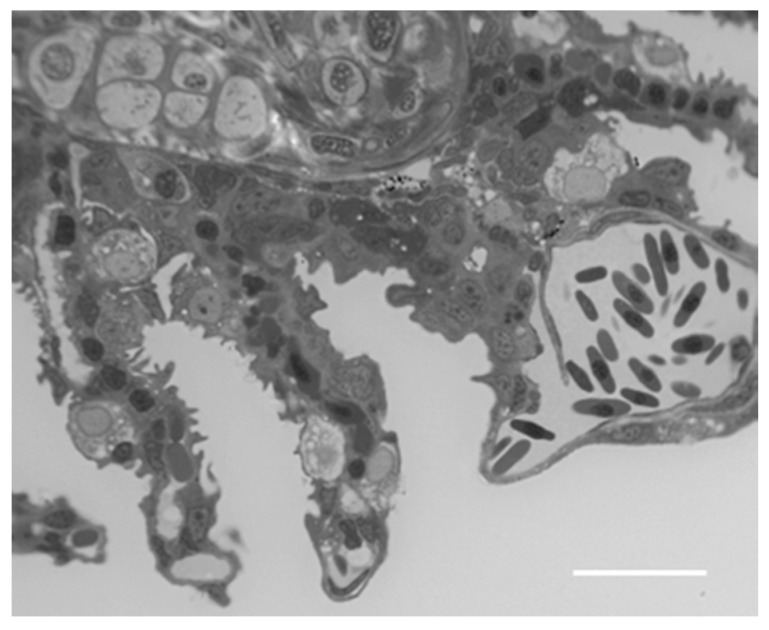
Cross section in toluidine blue of the gill apparatus in *D. rerio* after 96 h of exposure to 38.5 µg/L of HgCl_2_: hyperplasia of secondary epithelium with the appearance of CCs and MCs. Bar 20 µm.

From a histological point of view, we notice lamellar hyperplasia and the appearance of numerous CCs and MCs in the secondary lamellae (Figure 7).

**Figure 8:**
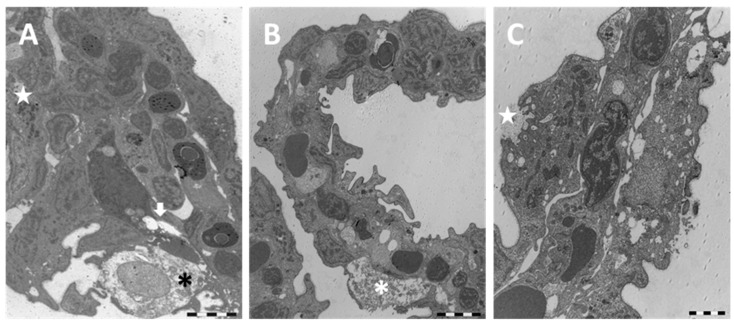
TEM micrographs of the gill apparatus in *D. rerio* after 96 h of exposure to 38.5 µg/L of HgCl_2_: (**A**) degeneration in primary epithelium with degeneration of mucous cells (asterisk), epithelial gaps (arrow), and macrophage infiltrations (star); (**B**) folding of secondary lamellae, hypertrophic mucous cell (asterisk), and tissue hyperplasia; (**C**) appearance of CCs in secondary lamellae and formation of deep invagination in CC (star). All bars 2 µm.

Under TEM, the profound degeneration of the gill apparatus is more evident. The epithelium of primary filaments is altered by the numerous intercellular gaps and by the massive degeneration of mucous cells; macrophage infiltrations are also frequently observed (Figure 8A).

The folding of the secondary lamellae is confirmed by ultrastructural observations that also reveal conspicuous hyperplasia phenomena (Figure 8B). In particular, the appearance of CCs is marked and some of these newly formed cells show deep apical invaginations (Figure 8C).

**Figure 9:**
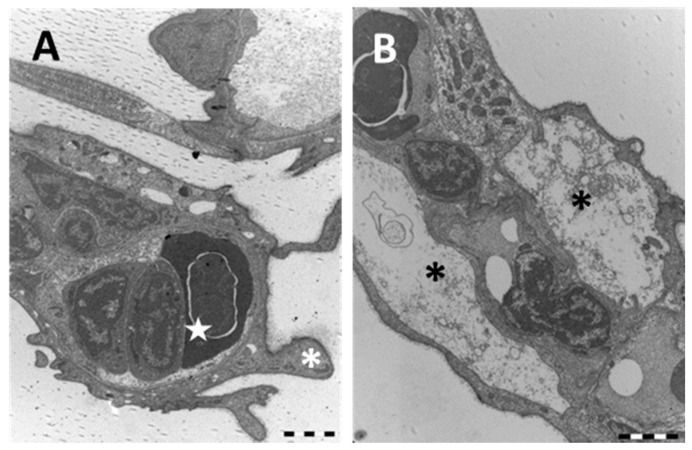
TEM micrographs of the gill apparatus in *D. rerio* after 96 h of exposure to 38.5 µg/L of HgCl_2_: (**A**) high magnification of long processes in PVCs (asterisks) and edema formation in the distal portion of lamellae (star); (**B**) degenerated and apoptotic PVCs (asterisks) with the disappearance of PCs. All bars 2 µm.

The whole epithelial surface shows an irregular profile due to long cytoplasmatic projection originating from the PVCs (Figure 9A). Blood congestion with the formation of aneurysms in the distal portions of lamellae often occur (Figure 9A). Cell degeneration is noticeable (Figure 9B) and in some areas the pillar cells also disappear.

### 2.2. Immunofluorescence and Real Time PCR

#### 2.2.1. MT

**Figure 10:**
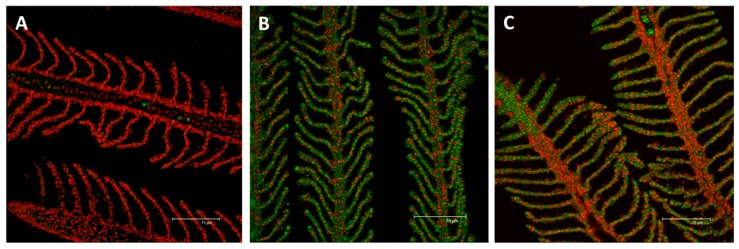
Confocal micrographs of the *D. rerio* gill apparatus. Sections labeled with a mouse monoclonal antibody against metallothionein (MT) (green–Fluorescein Isothiocyanate (FITC) labeled); nuclei labeled with propidium iodide (red); (**A**) no MTs expression in the gills of the control group; (**B**) after 96 h of exposure to 7.7 µg/L of HgCl_2_, MTs immunoreactivity strongly appear in both the primary and secondary epithelium; (**C**) after 96 h of exposure to 38.5 µg/L of HgCl_2_, the intensity of staining lightly decrease compared to the basal condition in both the filament and lamellar epithelium. All bars 75 µm.

The immunodetection for MTs shows the absence of signal in the gills of the animals from the control group (Figure 10A). After 96 hours of exposure to the low concentration of mercury chloride, it is possible to note a marked increase in MTs immunoreactivity, in both the filament and lamellae. The signal is mainly detected at the level of PVCs (Figure 10B). The localization pattern of MTs is similar in the samples from the high concentration group and also in this case, the signal is observed at the level of PVCs, in both the filament and lamellar epithelium (Figure 10C). However, the intensity of staining is lower than that observed in the low concentration group.

**Figure 11:**
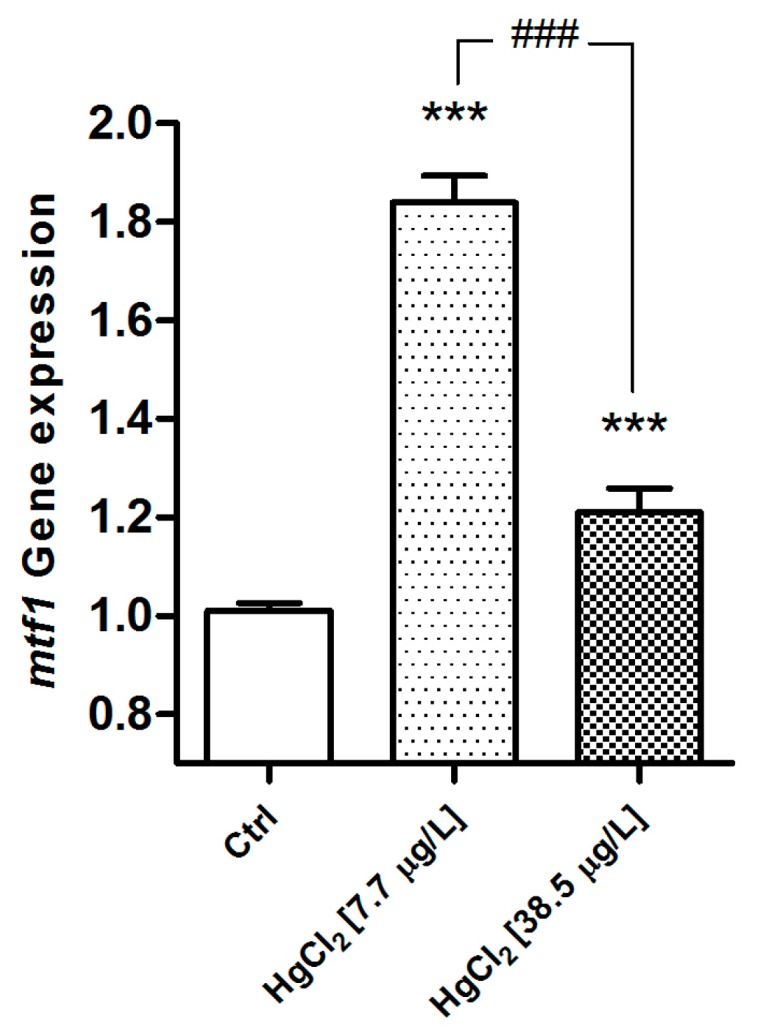
Relative variations in *mt* gene expression in the gill apparatus of *D. rerio* in the basal condition and after 96 hours of exposure to HgCl_2_ (7.7–38.5 µg/L). Taqman real time relative quantitative PCR. The bars show mean ± S.D, *n* = 5. Asterisks indicate the treated groups that differ from the control, *** *p* < 0.001; hashtags indicate difference between treated groups ^###^ *p* < 0.001 (One way ANOVA followed by Bonferroni’s post hoc test).

The genetic analysis confirms a dose-related expression rate. The gene shows a significant modification of *mt* expression after exposure to HgCl_2_ and the *mt* gene is significantly up-regulated in both experimental groups (*p* < 0.001), compared to the control. The gene shows the highest responses in the low concentration group (Figure 11). In the *D. rerio* gill apparatus, for both reference genes, no change has been reported after mercury exposure.

#### 2.2.2. Na^+^/K^+^-ATPase

**Figure 12:**
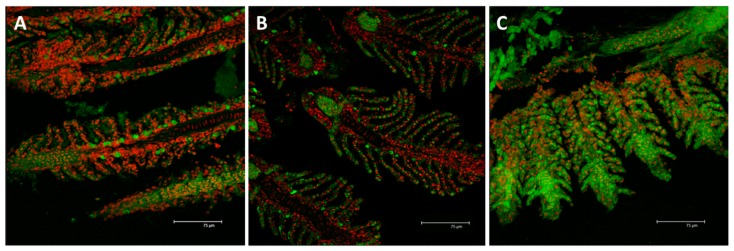
Confocal micrographs of *D. rerio* gill apparatus. Sections labeled with a mouse monoclonal antibody against Na^+^/K^+^-ATPase (green–FITC labeled); nuclei labeled with propidium iodide (red); (**A**) detection of Na^+^/K^+^-ATPase in the CCs of the interlamellar region in the basal condition; (**B**) after 96 h of exposure to 7.7 µg/L of HgCl_2,_ Na^+^/K^+^-ATPase immunoreactivity strongly decrease compared to the basal condition but the fluorescence labeling appears at the level of the secondary epithelium; (**C**) After 96 h of exposure to 38.5 µg/L of HgCl_2,_ the expression for Na^+^/K^+^-ATPase increase in both the filament and lamellar epithelium. All bars 75 µm.

In samples from the control group, the localization of Na^+^/K^+^-ATPase revealed immunopositive cells regularly distributed along the filament margins and in the interlamellar region; these cells are easily recognizable as CCs (Figure 12A). After exposure to the HgCl_2_ low concentration, the intensity and the distribution of the signal decrease compared to the control (Figure 12B).

On the contrary, after exposure to the high concentration of HgCl_2_, the Na^+^/K^+^-ATPase staining greatly increase compared to the basal conditions; moreover the signals are detected in both the filament and lamellar epithelium (Figure 12C).

**Figure 13:**
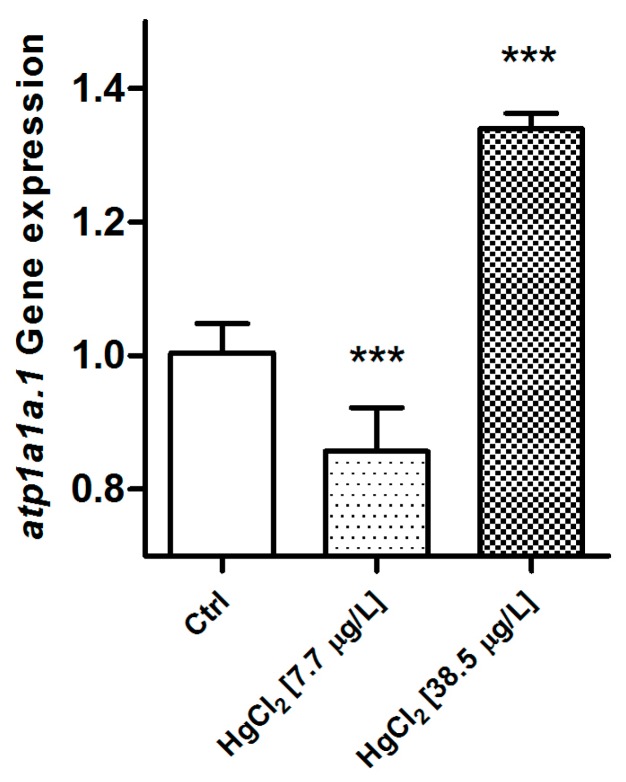
Relative variations in *atp1a1a.1* gene expression in the gill apparatus of *D. rerio* in the basal condition and after 96 hours of exposure to HgCl_2_ (7.7–38.5 µg/L). Taqman real time relative quantitative PCR. The bars show mean ± S.D, *n* = 5. Asterisks indicate the treated groups that differ from the control, *** *p* < 0.001 (One way ANOVA followed by Bonferroni’s post hoc test).

Also in this case, the genetic analysis reveals a significant modification after exposure to HgCl_2_. The gene expression level of *atp1a1a.1* is significantly down-regulated in the samples from the low concentration group when compared to the control group (*p* < 0.001). On the contrary, after exposure to the high concentration of HgCl_2_, the *atp1a1a.1* gene is significantly up-regulated compared to the basal condition (*p* < 0.001) (Figure 13). In the *D. rerio* gill apparatus, for both reference genes, no change has been reported after mercury exposure.

## 3. Discussion

The concept that fish gills are particularly sensitive to both the chemical and physical modification of surrounding water is widely accepted and morphofunctional alterations of fish gills after exposure to environmental pollutants have been widely documented. As such, it is necessary, however, to emphasize that the predictive role of gill lesions in ecotoxicological studies has been widely discussed and many authors suggest that gill damages are largely non-specific as they can be induced by a wide range of toxicants [43,44].

Several studies have been published on the effects of mercury on gill tissues, but most of the available data have been focused on methyl mercury (MeHg), whereas the effects of inorganic mercury are less investigated. Gill damages and structural changes caused by water-borne iHg have been reported for relatively few species, from both marine and freshwater ecosystems [24,25,26,27,45], and almost all previous studies have used a purely qualitative morphological approach [2].

The objective of the present paper is to provide valuable information for a more comprehensive understanding of mercury effects on fish gills by using a morphofunctional approach. In the present work, we clearly demonstrated that a short-term exposure to two low concentrations of mercury chloride resulted in gill morphology alterations and in the modifications of both Na^+^/K^+^-ATPase and MTs expression patterns in *D. rerio* gills. To the best of our knowledge, this is the first report describing morphofunctional and ultrastructural effects of iHg on zebrafish gills.

We recognized a wide variety of morphological alterations in *D. rerio* gills and our results are consistent with some previous findings on the effects of iHg on fish gills [24,26,27,45]. The severity of lesions observed in the gills of zebrafish became more pronounced with the increase of the pollutant dose resulting, at the high tested concentration, in the modification of both morphology and cell composition. The most common ultrastructural changes observed are represented by hyperplasia and ectopia of CCs, lamellar fusion, increased mucous secretion, ultrastructural alteration of PVCs, detachment of the secondary epithelium, pillar cells degeneration, degeneration, and apoptosis. Some of these alterations can easily interfere with the morphophysiology of this organ, impairing its normal function and preventing the exchange of gases and ions.

Hyperplasia of secondary epithelium often occur in the gills after exposure to heavy metals and it was frequently reported, in both freshwater fish and sea water fish, after exposure to mercury chloride and methylmercury [26,45,46]. One of the most frequent alterations observed in *D. rerio* was the occlusion of the interlamellar space, due to hyperplasia of the secondary epithelium that resulted in water flow disturbances. The hyperplasia of the epithelial cells could indicate an increase in cellular metabolism aimed to repair the sub-cellular damage or may be related to the cellular detoxification attempt which required, for instance, a greater synthesis of metallothionein [45]. However, the thickening of the secondary epithelium greatly increases the blood to water diffusion distances thus affecting gas and ion transfer, as previously reported in other species after exposure to Hg [27,47].

The epithelial surface of both the filament and lamellae is altered by the reduction and/or loss of PVCs apical microridges; these cytoplasmic folds, in basal conditions, mechanically enhance the surface for respiratory exchanges and it is supposed that they also play a role in osmotic regulation [48]. Jagoe and colleagues [24] suggest that the loss of microridges observed in the gills of *Gambusia holbrooki* after exposure to mercury chloride would be related to the swelling of pavement cells, thus reflecting the alterations in osmotic status of the surficial cells. In our work, we sometimes observed the lifting rather than the swelling of PVCs although the microridge alterations were an extensive phenomenon. Since the reduction of microridges has been previously reported after exposure to other toxicants [7], one may suggest that this may be a part of a protective effort to reduce the exchange with the surrounding water.

The attempt to counteract uptake of the pollutant is also accomplished by the enhanced mucous secretion, observed here after exposure to the high Hg concentration. A similar increase of mucus secretion has been observed in both freshwater fish and seawater fish after exposure to several heavy metals (Cu, Cd, Fe, and Ni) [37,49]. This great amount of mucous along with the reduction of apical microridges may induce hypoxia as observed in numerous fish species in different experimental conditions [7,50,51].

Beside the epithelial alterations, we also detected, in both concentrations groups, the disorganization of the lamellar blood space due to pillar cells’ degeneration. Alterations in pillar cells have been reported after exposure to both organic and inorganic mercury [26,52] and to other heavy metals [37,53,54,55]. The alteration of the cells controlling the blood contributes to the impairment of physiological exchange and also led to the appearance of aneurysms [7,9,26].

According to some authors, our results strongly support the hypothesis that the major cause of iHg toxicity would be the hypoxia or the loss of osmotic and ionic stability, although the mechanisms of uptake and the toxic action of dissolved inorganic Hg in gills need to be better clarified [2,24,26].

Other alterations observed in mercury exposed zebrafish are the detachment of the epithelium and cell degeneration. Epithelial sloughing off is one of the most common responses to pollutant exposure in freshwater fish [43,56]. This detachment of the epithelial surface has been previously reported in the secondary lamellae of the seawater fish *Dicentrarchus labrax* after exposure to 251, 355, and 501 µg/L of mercury [2], and in *Channa punctata* after exposure to a mixture of heavy metals [49]. Jagoe and colleagues [24] did not detected similar alterations in *Gambusia holbrooki* exposed to very low Hg concentrations, suggesting that this pathological response would be induced only by high doses of mercury. In our study, the detachment of the epithelium resulted from an exposure to two very low Hg concentrations, that correspond to 10% and 50% of the median lethal concentration at 96 h (LC5096) [57], thus leading us to hypothesize that the epithelial response to metals may also differ in different teleost species.

As stated above, in zebrafish the intensity of cellular injury increased with the mercury concentration and both apoptotic and necrotic cells were observed. Apoptosis and cell degeneration have been reported in fish gills as a consequence of chemical and physical perturbation of aquatic medium [2,45]. Mallatt [43] outlined that necrosis of the epithelium is more often associated with heavy metals in comparison to other toxicants and particularly with mercury. Such lesions have been detected in *Dicentrarchus labrax* (L.) after acute mercury exposure [2]. Daoust and colleagues [45] reported in *Oncorhynchus mykiss* the apoptosis in PVCs after 96 h of exposure to mercury chloride. The same authors suggest that the apoptotic events may play an important role in tissue homeostasis and may also be more advantageous compared to necrosis, avoiding tissue inflammation.

### 3.1. Chloride Cells

The teleost gill epithelium is characterized by the presence of chloride cells (CCs); these are large roundish cells typically distributed in the trailing edge of the filament epithelium and at the junctions between the filament and lamellae. Notwithstanding the differences among the various species, the ultrastructural distinctive features of the chloride cells are the numerous mitochondria scattered through the cytoplasm and the presence of an extensive tubular system originating from the basolateral membrane. These characteristics reflect their active role in ion transport and make the CCs the primary sites of active physiological processes in the gills [58].

One of the main effects of Hg exposure in zebrafish is the proliferation and the ectopia of CCs that inundates both lamellar epithelium and is responsible for the occlusion of interlamellar spaces. The hyperplasia of CCs is often reported under conditions that challenge ionic regulation such as exposure to toxicants [47] including heavy metals [49]. An increase in the number of CCs have been reported in the gill apparatus of *Dicentrarchus labrax* [2] and in *Gambusia holbrooki* [24] after exposure to mercury, and it is presumed that it may be related to the attempt of the epithelium to achieve ionic equilibrium.

Interestingly, along with the increase in number we also noted the appearance of an apical crypt in the newly formed CCs. In marine species, and in euryhaline species acclimated to seawater, CCs originate a multicellular complexes with accessory cells [57,58,59] and their membrane is typically provided with an apical crypt [60]. It has been recently suggested that the micro-environment which is created at the level of these deep apical invaginations plays a major role in determining ion uptake through the generation of a beneficial gradient and an enhancement of channel and transporter activity [61]. In addition, the polyanionic mucosubstance discovered in the apical crypts [62] seems to be involved in the concentration of cations and the maintenance of an ionic concentration gradient [60]. Therefore, on the basis of our data it is conceivable that both the enhancement in number and the rearrangement of the apical portion may play a major role in osmotic and ionic homeostasis.

### 3.2. Metallothioneins

The metallothioneins are non-enzymatic proteins with low molecular weight characterized by a high content of cysteine. The thiol groups (-SH) of cysteine residues allow MTs to bind heavy metals [63] and it was generally accepted that they also play a role in the homeostatic control of essential [34] and of some non-essential metals such as Cd and Hg [63,64]. Moreover, numerous studies have demonstrated the role of MTs in the detoxification of metals [63,65,66] and the correlation of MTs induction with an enhanced metal tolerance [34,63].

Due to their metal-binding properties, MTs are considered useful biomarkers of metal exposure, under both laboratory and field conditions [67]. The induction of MTs synthesis by heavy metals has been demonstrated in many teleost species, from both freshwater and seawater [68,69,70,71,72,73].

In teleost fish, the role of MTs in the gills is still contentious because of contradictory results. Some authors stated that gills would not be an useful target organ for MTs quantification [1,33,74] and MT responsiveness has been reported in the branchial epithelium of two seawater fish from mercury contaminated areas [67]. On the other hand, an increase in the expression levels of MTs has been observed in *Scatophagus argus* after only 72 h of exposure to mercury (30 µg/L) [75].

Our functional and molecular analysis revealed a significant increase in MTs expression after exposure to HgCl_2_ in *D. rerio* gills. However when comparing MTs levels in two tested concentrations, we can note that the highest response is detected in the low concentration group.

These results reflect the cellular detoxification attempt and are in line with the histopathological and ultrastructural alterations described above. In fact, in the present study, gills from the high concentration group showed conspicuous signs of epithelial degeneration, whereas in specimens from the low concentration group the gill morphology was still maintained; ultrastructural modification obviously interfere with protein biosynthesis.

### 3.3. Na^+^/K^+^-ATPase

According to the current model of transepithelial ion movements in the freshwater teleost gill [76,77], the chloride cells are the sites of the transport enzyme Na^+^/K^+^-ATPase [78]. Many studies reported that waterborne stressors are able to affect the Na^+^/K^+^-ATPase activity after both short and long time exposure to several stressors [7,79,80,81,82] and this enzyme is also considered a sensitive biomarker of heavy metal exposure [83].

Waterborne metals generally induce inhibition of Na^+^/K^+^-ATPase activity in gills, as reported in zebrafish [84] and in *Oreochromis niloticus* after exposure to copper [85]. Atli and Canli [86] reported in *Oreochromis niloticus* a decrease of expression after exposure to Cd, Cu, and Zn, but an increase after exposure to Pb. They explained this enhancement as an adaptation period dependent upon the continuing metal effect or maintenance of the ion flux [86].

Concerning mercury, a strong negative correlation between pollutant concentration and Na^+^/K^+^-ATPase activity has been reported in the gills of *Platichthys flesus* from a mercury contaminated area [31] and a similar inhibition has also been reported in *Cirrhinus mrigala* after an acute exposure to HgCl_2_ (0.068 and 0.034 mg/L) [29]. Jagoe and colleagues [28] demonstrated that there is no relationship between mercury environmental concentration and gill Na^+^/K^+^-ATPase activity in largemouth bass (*Micropterus salmoides*).

We clearly showed a significant level of Na^+^/K^+^-ATPase modulation in zebrafish gills from both mercury exposed groups compared to the control. In detail, we detected a decrease in Na^+^/K^+^-ATPase expression after exposure to the low tested concentration, but an increase after exposure to the high concentration. The significant increase demonstrated by RT-PCR in the high concentration group is confirmed by confocal analysis that revealed a great intensity of Na^+^/K^+^-ATPase labeling in all the CCs, including those situated on the lamellae. As stated above, in the high concentration group, the number of CCs greatly increased and these cells inundated the respiratory lamellae and conceivably, this may explain the enhanced expression of Na^+^/K^+^-ATPase. More studies are needed to better understand the reasons that underpin the variable responses observed in the expression of this enzyme in different fish species, metals, and exposure conditions [83].

## 4. Materials and Methods

### 4.1. Fish Maintenance and Experimental Set-Up

The experimental set-up has been previously described in detail [87], so a brief description will be given.

Specimens of *Danio rerio* used in our research (84 healthy adults of 6/8 months old of both sexes of length 0.35 ± 0.5 cm and weight 0.43 ± 0.06 g) were purchased from a local retailer. In the laboratory the animals were acclimated in two aquaria containing 80 L of dechlorinated water and were fed every two days before the beginning of the experiments. After two weeks, 14 animals for each experimental unit were transferred in aquaria of 30 L, containing two sub-lethal concentrations (7.7 and 38.5 µg/L) of mercury chloride (HgCl_2_, Sigma-Aldrich Chemical Co., St. Louis, MO, USA). Animals from the control group were maintained in aged tap water; each treatment was conducted in duplicate.

After 96 h the animals were anesthetized with tricaine methane sulfonate MS 222 (Sigma-Aldrich Chemical Co.) and the gills were taken from the animals. Animal care, experimental set-up, and killing were checked according to the European Convention for the Protection of Vertebrate Animals used for Experimental and other Scientific Purposes (Council of Europe No. 123, Strasbourg, 1985).

### 4.2. Light Microscopy and Electron Microscopy

After the gills were removal (for each experimental condition including the control, six animals were sacrificed for LM, TEM, and SEM), the samples were immersed in 4% glutaraldehyde (Electron Microscopy Sciences, Hatfield, PA, USA) in phosphate-buffered saline (PBS 0.1 M, pH 7.2, 4 °C) for 48 h. Samples were then post-fixed in osmium tetroxide (1% in PBS) for 2 h, dehydrated in graded ethanol, and then soaked in propylene-oxide.

Gill samples for light microscopy (LM) and transmission electron microscopy (TEM) were embedded in Epon-Araldite (Araldite 502/Embed 812, Electron Microscopy Sciences) and were cut using a Leica UltraCut UCT (Leica Microsystems, Wetzlar, Germany).

Semi-thin sections (1 µm) for light microscopy were stained with toluidine blue, and were then observed and photographed by a LM Leitz Dialux 20 EB (Leica Microsystems). Ultra-thin sections (800 Å) for TEM observations were stained with a uranyl acetate replacement, contrasted using lead citrate (Electron Microscopy Sciences), and were finally observed under a Zeiss EM 10 electron microscope (Zeiss, Oberkochen, Germany).

Samples for scanning electron microscopy (SEM) were dehydrated in ethanol and then dried in hexamethyldisilazane (HSDM), coated with gold in an Emitech K550 ion sputter unit (Quorum Technologies Ltd., The Broyle Ringmer, East Sussex, UK), and then observed using a FEI Quanta 200F scanning electron microscope (FEI company, Hillsboro, OR, USA).

### 4.3. Immunohistochemistry

For immunohistochemistry analysis, gill samples (for each experimental condition including the control, three animals were sacrificed) were fixed for 48 h by direct immersion in Bouin solution, dehydrated in an increasing series of ethanol solutions, cleared in xylene, and were finally embedded in paraffin wax with a mean fusion point of 56 °C. Tissue sections were cut (8 µm) and on deparaffinized slides the indirect immunofluorescence technique was applied [88].

The sections were washed in PBS and incubated in a moist chamber for 10 min with 20% normal goat serum (Sigma-Aldrich Chemical Co.). Some unwashed slides were incubated with a mouse monoclonal anti-metallothionein antibody (Stressgen Biotechnologies Corporation, Victoria, BC, Canada) and others with a mouse monoclonal anti Na^+^/K^+^-ATPase (Developmental Studies Hybridoma Bank, Iowa City, IA, USA) at working dilutions of 1:100. Next day slides were washed in PBS and incubated for 30 min at room temperature in the dark with fluorescein isothiocyanate-conjugated γ-globulin goat anti-mouse (1:50, Sigma-Aldrich Chemical Co.). After several washes in PBS, the sections were counterstained with propidium iodide for 30 s (Sigma-Aldrich Chemical Co.; 1:200 in PBS), washed again in PBS, and finally mounted.

A Leica TCS SP2 Confocal Laser Scanning Microscope (Leica Microsystems) was used for image acquisition.

### 4.4. Quantitative RT-PCR

The qPCR assays were performed according to the Minimum Information for Publication of Quantitative Real-Time PCR Experiments (MIQE) guidelines. Using the PureLink RNA Mini Kit (Thermo Fisher Scientific, Waltham, MA, USA) according to the manufacturer’s instructions, 30 mg of total RNA was extracted from the zebrafish gill tissue. An electrophoretic run on agarose-formaldehyde gel 1% was made to check the quality of the RNA produced and subsequently the RNA concentration was determined using a spectrophotometer. First-strand cDNA was synthesized from 2 µg of total RNA employing the High capacity cDNA RNA Kit (Applied Biosystems, Foster City, CA, USA) and the cDNA obtained was stored at −20 °C. Two different genes were used for this study: the metal-regulatory transcription factor 1 (*mtf1* NCBI Reference Sequence NM_152981.1) and the Na^+^/K^+^-ATPase pump (*atp1a1a.1*, NCBI Reference Sequence NM_131686.1); two reference genes were used: glyceraldehyde-3-phosphate dehydrogenase (*gapdh*, NCBI Reference Sequence NM_001115114.1) and actin beta 1 (*actb1*; NCBI Reference Sequence NM_131031.1).

The TaqMan Gene probe was used to check the amplification of the cDNA. Real-time PCR reactions (a cycle at 50 °C for 2 min, 95 °C for 10 min, and 40 cycles of amplification at 95 °C for 15 s, 60 °C for 1 min) were performed with a Light Cycler (Applied Biosystems Stepone, Real-Time PCR). The reaction mix (20 µL) contained: 2 µL of reverse transcribed product template, 10 µL of master mix (TaqMan Universal Master Mix II; Applied Biosystems), 1 µL of assay mix (TaqMan Gene Expression Assay; Applied Biosystems), and 7 µL of RNase-free H_2_O. For each experimental condition we performed five replicates and sacrificed five animals. The relative expression level quantification of the tested genes was normalized according to the mean in the expression levels of *gapdh* and *actb1*. The method 2^−Δ*C*t^ has been used to engender the relative mRNA expression of the gene [89].

### 4.5. Statistical Analysis

The effects of HgCl_2_ exposure on the expression levels of the target gene (*mtf1* and *atp1a1a.1*) were performed using Graph Pad Prism 5.00 (GraphPad Software Inc., San Diego, CA, USA) at a significance level of 0.05. The effects of mercury contamination were compared using one-way analysis of variance (one-way ANOVA), followed by Bonferroni’s Multiple Comparison Test.
